# Comparative Evaluation of Antioxidant Potential in Natural Plants, In Vitro Regenerants, and Callus Cultures of *Ungernia victoris* and *U. sewerzowii*

**DOI:** 10.3390/antiox15060763

**Published:** 2026-06-17

**Authors:** Hanifabonu Kobul kizi Juraeva, Abbos Tulkin ugli Khazratov, Feruza Usmanovna Mustafina, Madina Albertovna Shayakhmetova, Min Sung Lee, Chae Sun Na

**Affiliations:** 1 Tashkent Botanical Garden Named After Academician F.N. Rusanov of the Institute of Botany, Academy of Sciences, Tashkent 100053, Uzbekistan; hanifabonujurayeva@gmail.com (H.K.k.J.); xazratovabbos65@gmail.com (A.T.u.K.); mustafinaferuza@yahoo.com (F.U.M.); 2 Republican Scientific Center for Emergency Medical Care of the Ministry of Health, Tashkent 100081, Uzbekistan; madina250301@gmail.com; 3 Baekdudaegan National Arboretum of the Korea Forest Service (KFS), Bonghwa-gun 36209, Republic of Korea; next9101@koagi.or.kr

**Keywords:** *U. victoris*, *U. sewerzowii*, Amaryllidaceae, antioxidant activity, DPPH, ABTS, callus culture, in vitro regeneration, galantamine, plant tissue culture

## Abstract

*Ungernia victoris* and *U. sewerzowii* (Amaryllidaceae J.St.-Hil.) are rare medicinal species of Central Asia known as sources of biologically active alkaloids, including galantamine. In this study, antioxidant activity was comparatively evaluated across different types of plant material, including natural populations, botanical garden specimens, in vitro regenerants, callus cultures, in vitro obtained bulbs, and seeds. Micropropagation systems based on direct and indirect organogenesis were developed using Murasige and Skoog and Vollosovich et al. media with various plant growth regulator combinations. Antioxidant activity was determined with the use of DPPH and ABTS assays and expressed as IC_50_ values. Significant variability was observed depending on population origin, type of biological material, and in vitro cultivation conditions. *U. sewerzowii* demonstrated higher antioxidant activity than *U. victoris* in natural populations. The highest activity was recorded in callus cultures, whereas in vitro-derived bulbs showed relatively low activity. A strong positive correlation between DPPH and ABTS assays confirmed the reliability of the results and indicated the contribution of multiple types of secondary metabolites. These findings highlight the potential of *Ungernia* callus cultures as a promising biotechnological platform for the production of antioxidant-active compounds and support sustainable utilization strategies.

## 1. Introduction

In recent years, there has been a growing interest in natural sources of antioxidant compounds due to their important role in the prevention and treatment of diseases associated with oxidative stress. Reactive oxygen species (ROS), including superoxide anion, hydroxyl radical, and hydrogen peroxide, are natural by-products of cellular metabolism [1]. However, their excessive accumulation can lead to damage of lipids, proteins, and nucleic acids. Oxidative stress, caused by an imbalance between excessive ROS production and insufficient antioxidant defense, is associated with numerous pathologies, including aging-related diseases, cancer, cardiovascular, inflammatory, and neurodegenerative disorders such as Parkinson’s disease and Alzheimer’s disease [2,3,4]. According to the “free radical theory of aging,” proposed by Denham Harman in 1956, the harmful effects of ROS generated during mitochondrial respiration play a direct role in the aging process [5].

Antioxidants play a crucial role in protecting cells from oxidative damage. The use of antioxidants and activity modulators for the prevention and treatment of brain pathologies has attracted significant attention from the scientific community. Studies indicate that these compounds are capable of protecting neuronal cells from oxidative damage and reducing inflammation, which is one of the main factors contributing to the development of many neurological disorders [6,7,8]. In recent years, particular attention has been paid to plant-derived natural antioxidants, which exhibit high biological activity and relatively low toxicity. Many medicinal plants contain a wide range of secondary metabolites, including phenolic compounds, flavonoids, alkaloids, and terpenoids, which are characterized by pronounced antioxidant properties. Medicinal plants are considered an accessible and potent source of antioxidants, as they contain a complex mixture of chemical compounds that may act individually or synergistically to prevent disease and improve human health [9,10,11,12,13].

Plants of the family *Amaryllidaceae* J.St.-Hil. represent one of the most promising sources of biologically active compounds. This family includes more than 80 genera and approximately 1600 species widely distributed across different regions of the world [14,15]. Members of this family are characterized by a high content of specific isoquinoline alkaloids with diverse pharmacological activities. To date, more than 600 Amaryllidaceae alkaloids have been identified, among which galantamine, lycorine, homolycorine, and tazettine are of particular importance.

The genus *Ungernia* Bunge represents an important group of plants within the Amaryllidaceae family, predominantly distributed in Central Asia. Several species of this genus are endemic to mountainous regions and possess significant pharmacological potential [16,17].

The total alkaloid content in *U. victoris* varies depending on habitat and climatic conditions [18,19]. The total alkaloid content in leaves and bulbs ranges from approximately 0.5 to 0.52%. The proportion of galantamine in the total alkaloid mixture reaches about 0.13–0.15% in dried leaves. Approximately ten different alkaloids have been identified in this species. In addition to galantamine, dried leaves of *U. victoris* contain lycorine (0.073%), hordenine (0.039%), tazettine (0.1%), pancratinine (0.15%), and dl-narwedine (0.0054%) [20,21,22,23].

Galantamine is one of the most well-known Amaryllidaceae alkaloids and is widely used in the pharmacotherapy of Alzheimer’s disease as an acetylcholinesterase inhibitor [9,10].

Leaves of *U. sewerzowii* contain important biologically active compounds, with lycorine being the predominant component (0.04% in leaves and 0.15% in bulbs from Burchmulla populations in Uzbekistan, and up to 0.46% in leaves and 0.38% in bulbs from Karzhantau populations). According to Smirnova et al. (1964), *U. sewerzowii* contains pancratinine (0.12% in leaves and 0.64% in bulbs), galantamine (0.01% in leaves and 0.033% in bulbs), dl-narvezine (0.03% in leaves and 0.01% in bulbs), hippeastrine (0.0008% in leaves and 0.0002% in bulbs), ungeremine (0.0005% in leaves and 0.074% in bulbs), tazettine (0.0001% in leaves and 0.1% in bulbs), ungerine (0.043% in leaves and 0.089% in bulbs), and unsevine (0.016%, detected only in roots) [24]. Lycorine is used in medicine for the treatment of acute and chronic bronchitis and exhibits pronounced emetic and expectorant properties. Currently, lycorine hydrochloride is applied as an expectorant in inflammatory diseases of the lungs and bronchi, as well as in bronchial asthma.

Despite the pharmacological importance of these species, data on their antioxidant activity remain limited. Most studies on *Ungernia* species have primarily focused on alkaloid composition and pharmacological activity of individual compounds. At the same time, the antioxidant potential of different types of plant material has not been sufficiently investigated.

In recent years, plant cell and tissue culture has been considered a promising alternative to the traditional harvesting of plant raw materials from natural populations. This is particularly relevant under conditions of decreasing natural resources, as intensive harvesting, anthropogenic impact, and environmental changes lead to a reduction in population size and even extinction of many species in their natural habitats. In some cases, lost natural populations cannot be restored, making in vitro methods not only an efficient approach for obtaining valuable secondary metabolites but also an important tool for the conservation of genetic resources of rare and endemic plant species. This approach enables the production of valuable secondary metabolites under controlled conditions, independent of seasonal and environmental factors [25,26].

Therefore, the aim of the present study was a comparative analysis of the antioxidant activity of different types of plant material of two *U.* species—*U. victoris* and *U. sewerzowii*. The study included plants from natural populations, botanical garden specimens, in vitro regenerated plants, callus cultures, and seeds.

Antioxidant activity was evaluated using DPPH (2,2-diphenyl-1-picrylhydrazyl) and ABTS (2,2′-azino-bis(3-ethylbenzothiazoline-6-sulfonic acid)) radical scavenging assays. The obtained results allow identification of the most promising sources of antioxidant compounds among different types of plant material and expand current knowledge on the biochemical potential of *Ungernia* species.

## 2. Materials and Methods

### 2.1. Plant Material

The objects of the study were two medicinal species of the genus *Ungernia* Bunge—*U. victoris* Vved. ex Artjush. and *U. sewerzowii* (Regel) B.Fedtsch. (*Amaryllidaceae* J.St.-Hil.)—distributed in the mountainous regions of Central Asia and of considerable interest as sources of biologically active compounds, primarily Amaryllidaceae alkaloids [27].

*U. victoris* is a perennial bulbous herbaceous plant with an ovoid bulb measuring approximately 7–12 cm in diameter, covered with dark brown or blackish membranous scales. The leaves are linear, arranged in a basal rosette, up to 20–25 cm long, with a glaucous tint. The inflorescence is umbellate, with actinomorphic flowers bearing six perianth segments. The fruit is a dry capsule [27,28].

*U. sewerzowii* is also a perennial bulbous herbaceous plant. The bulb is ovoid or rounded and covered with brownish membranous scales. The leaves are linear or linear-lanceolate, forming a basal rosette of a glaucous-green color. The leafless scape bears an umbellate inflorescence consisting of several flowers. The flowers are actinomorphic with six perianth segments of pinkish or reddish coloration. The fruit is a dry capsule containing numerous seeds [27,28].

**Figure 1:**
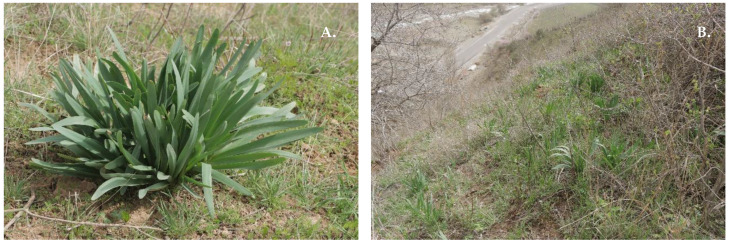
*Ungernia victoris* Vved. ex Artjush. (**A**) Adult plant. (**B**) Population UV1. Habitat: Polgasay River valley, vicinity of the Sangardak waterfall, Hissar Range, Surkhandarya region, Uzbekistan. 10 April 2023. Photo by D. Turdiev.

**Figure 2:**
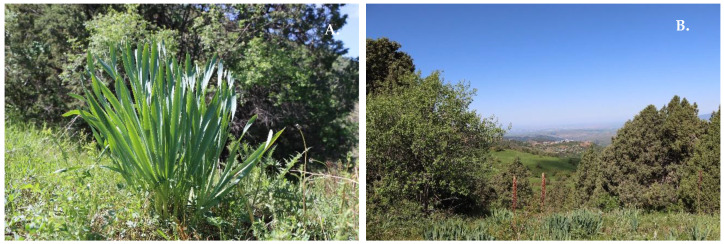
*Ungernia sewerzowii* (Regel) B.Fedtsch. (**A**) Adult plant. (**B**) Population US5. Beldersay River valley, vicinity of the meteorological station, Chatkal Range, Western Tien Shan, Tashkent region, Uzbekistan. 7 June 2021. Photo by D. Turdiev.

Both species are distributed in the mountainous regions of Central Asia. *U. victoris* is an endemic species occurring mainly in Uzbekistan and Tajikistan (Figure 1A,B), whereas *U. sewerzowii* has a wider distribution and is found in Uzbekistan and southern Kazakhstan (Figure 2A,B). The plants grow on rocky and gravelly slopes, predominantly within the middle mountain belt at altitudes ranging from 600 to 2700 m above sea level, forming local populations.

Information on the sampling sites of seeds and bulbs of the studied *Ungernia* species is presented in Table 1. Plant material was collected from natural populations of *U. victoris* and *U. sewerzowii* in mountainous regions of Central Asia (Uzbekistan).

Populations of *U. victoris* were studied in the Surkhandarya region within the Hissar Range of the Pamir-Alai mountain system, while populations of *U. sewerzowii* were investigated in the Western Tien Shan (Tashkent region). For each population, geographic coordinates, altitude, sampling date, and field sample codes were recorded (Table 1).

The collected plant material was used for subsequent experiments, including the establishment of in vitro cultures, callus lines, and the evaluation of antioxidant activity of different types of plant material.

In this study, various types of plant material of *U. victoris* and *U. sewerzowii* were used, including plants from natural populations, plants cultivated under botanical garden conditions, in vitro regenerated plants, callus cultures, and seeds. The use of different types of plant material enabled a comparative analysis of antioxidant activity and assessment of the influence of cultivation conditions on the accumulation of biologically active compounds.

### 2.2. Methods

The protocols for seed sterilization, explant selection, micropropagation, callus induction, and regeneration of *U. victoris* used in the present study were previously developed and described in detail by Mustafina et al. (2024) [29]. The present investigation did not aim to optimize culture conditions or evaluate plant growth regulator combinations. Instead, antioxidant activity was assessed using plant materials and callus lines obtained through the previously established and optimized protocols. Therefore, only the culture media and biological materials relevant to the antioxidant and phytochemical analyses are presented herein, while detailed descriptions of sterilization procedures, explant selection criteria, and medium optimization are available in the cited publication.

### 2.3. Nutrient Media

Murashige and Skoog (MS) medium (1962) [30] was obtained as a ready-made formulation from Duchefa Biochemie B.V. (Haarlem, The Netherlands). Vollosovich et al. medium (Vch) (1979) [31] was prepared according to the original formulation using analytical-grade chemical components supplied by the same manufacturer. The culture media were selected based on previous optimization experiments and published protocols [29,32]. Solid media were supplemented with agar–agar Roko Agar (Llanera, Spain) as a gelling agent.

The MS medium is characterized by a high content of macronutrients, particularly nitrate nitrogen. The concentrations of the main macronutrients were as follows: NH_4_NO_3_—1650 mg/L, KNO_3_—1900 mg/L, MgSO_4_·7H_2_O—370 mg/L, KH_2_PO_4_—170 mg/L, CaCl_2_·2H_2_O—440 mg/L. The micronutrient composition corresponded to the standard MS formulation. Sucrose (30 g/L) was used as a carbon source, and agar (7 g/L) as a gelling agent. The pH of the medium was adjusted to 5.6–5.8 prior to sterilization.

The Vch medium differs significantly by having a lower nitrate content and a different macronutrient ratio. The concentrations of the main components were: NH_4_NO_3_—600 mg/L, KNO_3_—1200 mg/L, MgSO_4_·7H_2_O—600 mg/L, Ca(NO_3_)_2_·4H_2_O—1000 mg/L, NH_4_H_2_PO_4_—700 mg/L, (NH_4_)_2_SO_4_—400 mg/L, and KCl—80 mg/L. Additionally, the medium contained organic components stimulating morphogenesis, including myo-inositol (100 mg/L), glycine (2.5 mg/L), and casein hydrolysate (500 mg/L). The sucrose concentration was 50 g/L, agar—7 g/L, and pH was adjusted to 5.6–5.8 with pH-meter 200E (Jinan Xinghua Instruments Co., Ltd., Jinan, China).

Thus, the main difference between the two media lies in the reduced nitrate nitrogen content and the presence of additional organic components in the Vch medium, which promote callus formation and organogenesis. In this study, MS medium was primarily used for seed germination and initial explant establishment, whereas Vch medium was applied during callus induction, organogenesis, and plant regeneration stages of *U. victoris* and *U. sewerzowii*. In addition, callus tissues obtained on MS medium were also evaluated for antioxidant activity.

### 2.4. Plant Growth Regulators

To induce callus formation, organogenesis, and plant regeneration in *U. victoris* and *U. sewerzowii*, various combinations of plant growth regulators were added to the culture media. Auxins included 2,4-dichlorophenoxyacetic acid (2,4-D), indole-3-acetic acid (IAA), and α-naphthaleneacetic acid (NAA), while cytokinins included 6-benzylaminopurine (BAP), kinetin (Kin), and zeatin (Zea). The concentrations of plant growth regulators ranged from 0.5 to 4.0 mg/L. All growth regulators were obtained from Duchefa Biochemie B.V. (Haarlem, The Netherlands).

In a previous study by Mustafina et al. (2024) [29], 183 combinations of plant growth regulators differing in type and concentration were screened, and the most effective media for callus induction and regeneration were selected. The present study utilized plant material obtained using these optimized culture conditions. For convenience, the following abbreviations were used: M—Murasige and Skoog medium, V—Vollosovich medium. Accordingly, different medium variants were designated as M or V followed by a numerical code (e.g., M17, M32, V56, V68), reflecting specific combinations of growth regulators used at different stages of micropropagation.

### 2.5. Protocols of Micropropagation of U. victoris and U. sewerzowii

Explants were obtained from germinated seedlings approximately two weeks after seed germination on nutrient media. The hypocotyl segment of germinated seeds was used as the explant according to previously published work [29]. Each treatment was evaluated in at least three independent replicates, with 20–30 explants per treatment. Detailed information regarding explant numbers and experimental design is provided in Mustafina et al. (2024) [29].

Seeds were collected in September and stored at +5 °C in refrigerator BPR5V628F (Biobase, Jinan, China) for two weeks. They were then cleaned from impurities and thoroughly washed under running water. Subsequently, seeds were treated in a soap solution under constant agitation (150 rpm) on SK-0330 Pro orbital shaker (DLAB Scientific Co., Ltd., Beijing, China) for 20 min and rinsed with distilled water.

Surface sterilization was performed using 4% sodium hypochlorite solution for 10 min, followed by thorough rinsing with distilled water. A total of 25–30 sterilized seeds were placed in Petri dishes containing 25% MS medium supplemented with sucrose (7 g/L) and agar (7 g/L), without plant growth regulators. Each Petri dish (Minimed, Suponevo, Russia) contained 25 mL of medium.

The Petri dishes were sealed with stretch film and kept at +5 °C in refrigerator BPR5V628F (Biobase, Jinan, China) for two weeks. After stratification, the seeds were transferred to 50% MS medium in 0.5 L glass vessels containing sucrose (15 g/L) and agar (7 g/L), without growth regulators, and incubated in a growth room at 24 ± 2 °C, relative humidity of 40%, and illumination of 1300 lux. After three weeks, germinated seeds were used as a source of explants, with the hypocotyl showing the highest efficiency.

Micropropagation was carried out using in vitro tissue culture techniques on modified MS and Vch media [30,31,32,33,34,35]. Explants were introduced into culture on media supplemented with various combinations of plant growth regulators. The most effective media for callus induction contained 2,4-D at a concentration of 0.5 mg/L in combination with cytokinins.

#### 2.5.1. Micropropagation via Indirect Organogenesis in *U. victoris* and *U. sewerzowii*

**Figure 3:**
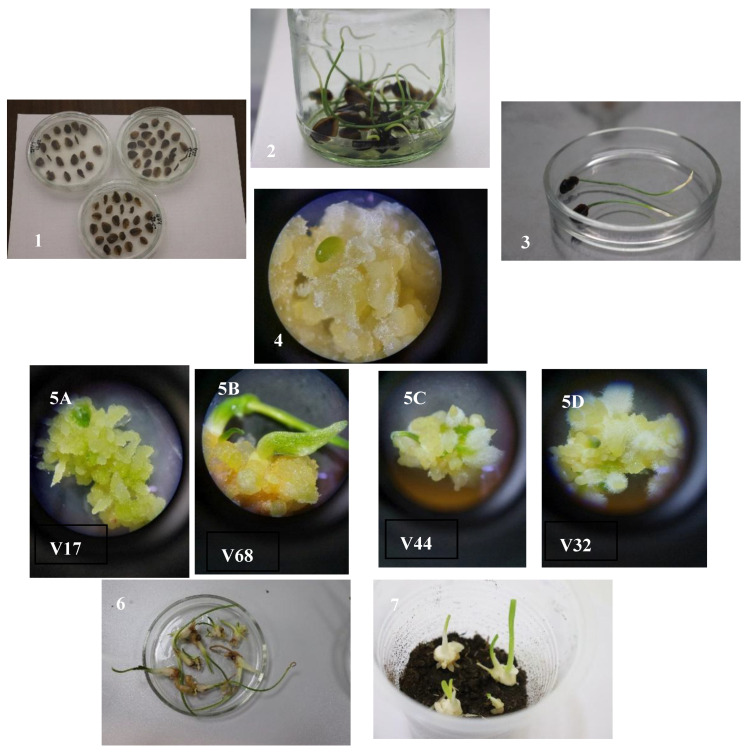
Scheme of indirect organogenesis in two *Ungernia* species: *U. victoris* and *U. sewerzowii*. 1. Stratification of freshly collected seeds at +5 °C on 25% Murasige and Skoog (MS) medium in Petri dishes for two weeks. 2. Transfer of stratified seeds into 0.5 L vessels containing 50% MS medium and cultivation in a growth room at +24 ± 2 °C. 3. Use of germinated seeds as a source of explants for culture initiation. 4. Callus development on Vollosovich et al. (Vch) medium supplemented with plant growth regulator combinations (2.4D + BAP; 2.4D + Kin). 5. Different morphogenetic responses of callus on Vch media: (A) V17 (IAA 0.5 mg/L + BAP 0.5 mg/L): hemmogenesis (microbulb formation) with weak root system development. (B) V32 (NAA 0.5 mg/L + BAP 0.5 mg/L): hemmorhizogenesis, with shoot formation prevailing over rhizogenesis. (C) V44 (NAA 0.5 mg/L + Kin 0.5 mg/L): intensive hemmorhizogenesis with active development of root hairs and root elongation. (D) V68 (IAA 0.5 mg/L + Kin 0.5 mg/L): intensive microbulb formation, while root development proceeds more slowly. 6–7. Microbulbs corresponding morphologically to 2–3-year-old bulbs under natural conditions.

For both species, two regeneration pathways were observed: indirect organogenesis (Figure 3) via a callus phase and direct organogenesis without callus induction [29,36].

For callus induction in *U. victoris* hypocotyl explants, the most effective results were obtained using the following combinations of plant growth regulators: V5 (2,4-D 0.5 mg/L + BAP 0.5 mg/L), V56 (2,4-D 0.5 mg/L + Kin 0.5 mg/L), and V162 (BAP 0.5 mg/L).

For callus induction in *U. sewerzowii* hypocotyl the most effective results were obtained using the following combinations of plant growth regulators: V5 (2,4-D 0.5 mg/L + BAP 0.5 mg/L), V56 (2,4-D 0.5 mg/L + Kin 0.5 mg/L), and V87 (2,4-D 0.5 mg/L + Zea 0.5 mg/L).

The first subculture of callus-derived explants was carried out on the same media used for culture initiation. For *U. victoris*, these included V5, V56, and V162, while for *U. sewerzowii*, V5, V56, and V87 were used. The second subculture stage, aimed at mass propagation, was performed on medium M56 (2,4-D 0.5 mg/L + Kin 0.5 mg/L).

At this stage, intensive callus proliferation was observed, with callus formation initiating in the basal region of the hypocotyl in approximately 80 ± 2% of explants. Initially, the callus tissue exhibited a bright yellow coloration, which gradually changed to a greenish hue.

Subsequently, callus tissues of both species were transferred to regeneration media: V17 (IAA 0.5 mg/L + BAP 0.5 mg/L), V32 (NAA 0.5 mg/L + BAP 0.5 mg/L), V44 (NAA 0.5 mg/L + Kin 0.5 mg/L), and V68 (IAA 0.5 mg/L + Kin 0.5 mg/L). After one month, subculturing was performed on Vch medium with an increased auxin-to-cytokinin ratio (5:1) to stimulate microbulb formation: V17 (IAA 2.5 mg/L + BAP 0.5 mg/L), V32 (NAA 2.5 mg/L + BAP 0.5 mg/L), V44 (NAA 2.5 mg/L + Kin 0.5 mg/L), and V68 (IAA 2.5 mg/L + Kin 0.5 mg/L).

Further subculturing was carried out on 50% Vch medium to stimulate root development, supplemented with NAA (2.5 mg/L), BAP (0.5 mg/L), and TDZ (0.3 mg/L). Subsequent subcultures were maintained on Vch medium containing NAA (0.5 mg/L), which promoted root formation and acclimatization readiness.

Within six months, up to 100–150 microbulbs were produced per explant, corresponding morphologically to two-year-old bulbs formed under natural conditions.

#### 2.5.2. Micropropagation via Direct Organogenesis in *U. victoris* and *U. sewerzowii*

Direct organogenesis was observed when hypocotyl explants were cultured directly on MS or Vch media without an intermediate callus phase. The most effective regeneration was achieved on Vch medium variants V17, V32, V44, and V68 [35].

After one month, explants were transferred to media with an increased auxin-to-cytokinin ratio (5:1) to induce microbulb formation: V17, V32, V44, and V68 with elevated auxin concentrations (2.5 mg/L).

Root development was subsequently stimulated on 50% Vch medium supplemented with NAA (2.5 mg/L), BAP (0.5 mg/L), and TDZ (0.3 mg/L). Further subculturing was carried out on Vch medium with NAA (0.5 mg/L), facilitating root system development and preparation for ex vitro acclimatization.

Under these conditions, 2–3 microbulbs were formed per explant. These microbulbs corresponded morphologically to two-year-old bulbs, characterized by 2–3 well-developed true leaves and a functional root system.

### 2.6. Preparation of Plant Material for Chemical Analysis

Various types of plant material from *U. victoris* and *U. sewerzowii* were used, including plants from natural populations, botanical garden specimens, in vitro regenerated plants, callus cultures, and seeds. In vitro-derived regenerants and callus cultures were analyzed under different culture conditions, including MS and Vch media supplemented with various combinations of plant growth regulators. Collected plant material was cleaned from impurities and dried in a drying oven ShS 80-01 (SKTB SPU, Smolensk, Russia) at 40 °C until constant weight. Dried samples were ground manually into a homogeneous powder.

### 2.7. Phytochemical Characterization (HPLC Analysis)

HPLC analysis was performed using an Agilent 1260 Infinity system (Agilent Technologies, Santa Clara, CA, USA) equipped with a reversed-phase column ZORBAX Eclipse Plus C18 (4.6 × 250 mm, 5 μm; Agilent Technologies, Santa Clara, CA, USA). The mobile phase consisted of (A) 0.1% trichloroacetic acid in water and (B) 0.1% trichloroacetic acid in acetonitrile. Gradient elution was applied as follows: 0 min, 97% A/3% B; 7 min, 95% A/5% B; 30 min, 50% A/50% B; and 50 min, 0% A/100% B. The flow rate was set at 1.0 mL/min, the injection volume was 10 μL, and detection was carried out at 288 nm. The total run time was 50 min. Galantamine was identified by comparing its retention time with that of an authentic standard. The retention time of galantamine under these conditions was approximately 2.3–2.4 min.

### 2.8. DPPH Radical Scavenging Assay

Antioxidant activity was evaluated using the DPPH (2,2-diphenyl-1-picrylhydrazyl) radical scavenging assay according to Brand-Williams et al. (1995) [37], with minor modifications.

A 0.1 mM DPPH solution was prepared in methanol. One milliliter of the DPPH solution was mixed with 1 mL of plant extract at different concentrations. The reaction mixture was thoroughly vortexed and incubated in the dark at room temperature for 30 min.

The absorbance was measured at 517 nm using a Cary 60 UV–Vis spectrophotometer (Agilent Technologies, Santa Clara, CA, USA). The decrease in absorbance was used to determine the radical scavenging activity of the extracts. Antioxidant activity was expressed as IC_50_ values, defined as the concentration required to inhibit 50% of DPPH radicals.

Ascorbic acid was used as a reference antioxidant (positive control) to enable comparison of the antioxidant capacities of the tested samples with a well-established antioxidant standard. Under the experimental conditions, the IC_50_ value of ascorbic acid in the DPPH assay was 17.21 μg/mL.

All measurements were performed in triplicate, and the results were expressed as mean ± standard deviation.

### 2.9. ABTS Radical Scavenging Assay

Antioxidant activity was also determined using the ABTS radical cation decolorization assay according to Re et al. (1999) [38].

The ABTS radical cation solution was prepared by mixing 7 mM ABTS with 2.45 mM potassium persulfate and incubating the mixture in the dark at room temperature for 12–16 h to generate a stable radical cation.

Prior to use, the solution was diluted with ethanol to obtain an absorbance of 0.70 ± 0.02 at 734 nm. A volume of 10–50 μL of plant extract was added to 1 mL of ABTS solution. After 6 min of incubation at room temperature, absorbance was measured at 734 nm using a Cary 60 UV–Vis spectrophotometer (Agilent Technologies, Santa Clara, CA, USA). Antioxidant activity was expressed as IC_50_, defined as the concentration required to scavenge 50% of ABTS radicals. All experiments were conducted in triplicate.

Ascorbic acid was used as a reference antioxidant (positive control). Under the experimental conditions, the IC_50_ value of ascorbic acid in the ABTS assay was 8.33 μg/mL.

### 2.10. Statistical Analysis

All experiments were performed in triplicate, and the results are presented as mean values ± standard deviation (SD). Statistical analyses were performed using Microsoft Excel 2019 Analysis ToolPak (Microsoft Corporation, Redmond, WA, USA). Differences among treatments were evaluated using one-way ANOVA at *p* < 0.05. One-way ANOVA was selected because population origin, biological material type, and culture medium effects were analyzed independently rather than within a fully balanced multifactorial design. Differences were considered statistically significant at *p* < 0.05.

Correlation analysis between DPPH and ABTS antioxidant activity values was performed using Pearson’s correlation coefficient (r). The strength of correlation was interpreted according to standard criteria.

## 3. Results and Their Discussion

### 3.1. Micropropagation of U. victoris and U. sewerzowii

Both species exhibited direct and indirect organogenesis, with the morphogenetic response varying depending on the composition of plant growth regulators. Examples of indirect organogenesis are shown in Figure 3.

V17 (IAA 0.5 mg/L + BAP 0.5 mg/L). Hemmogenesis, i.e., microbulb formation, was observed, whereas root system development remained weak. The resulting microbulbs were characterized by well-developed leaves reaching 7–8 cm in length.

V32 (NAA 0.5 mg/L + BAP 0.5 mg/L). Hemmorhizogenesis was observed, with shoot formation proceeding more intensively than rhizogenesis. Up to 100–150 microbulbs were formed per explant. At the initial stages, the callus tissue was light yellow, later becoming dark yellow or green. Dark-yellow buds developed on the callus, subsequently turning green, elongating, and developing into microbulbs. Rapid leaf development was recorded, whereas root formation proceeded relatively slowly. Compared with medium V17, root system development was more intensive on this medium.

V44 (NAA 0.5 mg/L + Kin 0.5 mg/L). After transfer of the callus to this medium, intensive hemmorhizogenesis was observed compared with media V17, V32, and V68. Active development of root hairs and root elongation were noted. Up to 100–150 microbulbs with a well-developed root system were formed per explant. Bulbs transferred to this medium produced developed roots, which ensured successful adaptation to soil conditions.

V68 (IAA 0.5 mg/L + Kin 0.5 mg/L). This medium stimulated intensive microbulb formation; however, root system development was slower. Approximately 100–150 microbulbs were formed per explant.

Indirect organogenesis in *U. victoris* and *U. sewerzowii*, observed on Vch medium using the hypocotyl of germinated seeds as explants, resulted in the formation of 100–150 microbulbs per explant. In contrast, during direct organogenesis, the number of developing microbulbs was significantly lower, averaging 2–3 microbulbs per explant. The morphogenetic response of explants to different combinations of plant growth regulators in MS media was similar under both direct and indirect organogenesis. In both species, the period from explant introduction into in vitro culture to plant adaptation under soil conditions was 5–6 months.

### 3.2. Direct Organogenesis Proceeded Without Callus Formation

V17 (IAA 0.5 mg/L + BAP 0.5 mg/L). No callus formation was observed on this medium. However, 2–3 microbulbs formed directly on the explants without a callus stage, with a weakly developed root system or no roots.

V32 (NAA 0.5 mg/L + BAP 0.5 mg/L). Callus formation was observed in 10 ± 2% of explants, and the callus was light yellow in color. As a result of direct organogenesis, well-developed microbulbs were formed; however, the root system was weakly developed or absent. In some explants, 2–3 microbulbs were formed per explant.

V44 (NAA 0.5 mg/L + Kin 0.5 mg/L). Callusogenesis was observed in 40 ± 2% of explants, and the callus had a light-yellow coloration. This combination of growth regulators stimulated root system development. Small microbulbs formed directly on the explants or on the callus tissue, accompanied by intensive root formation with numerous root hairs. Each explant produced 2–3 microbulbs.

M68 (IAA 0.5 mg/L + Kin 0.5 mg/L). Weak callus formation was recorded only in 10 ± 2% of explants, and the callus was light yellow. Each explant produced 2–3 microbulbs, which were nevertheless comparatively well developed.

### 3.3. Analysis of the Antioxidant Activity of Different Types of Plant Material of U. victoris and U. sewerzowii

1. Intraspecific Variability of Antioxidant Activity in *U. victoris*.

**Figure 4:**
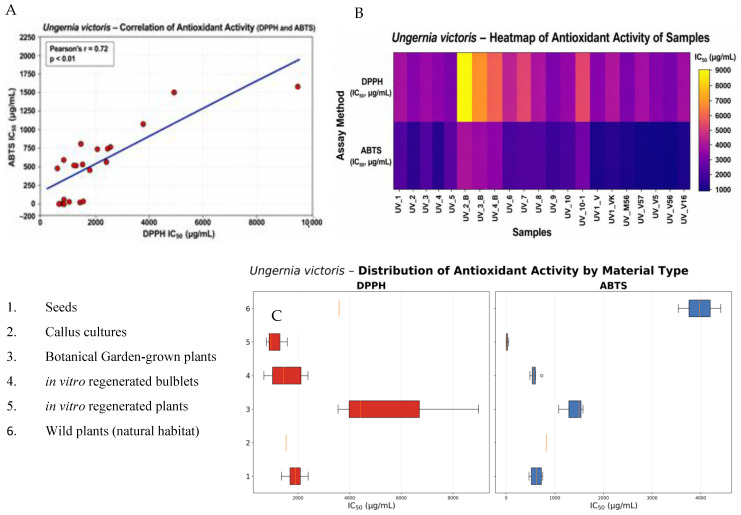
Antioxidant activity of different types of plant material of *Ungernia victoris*. (**A**) Correlation between DPPH and ABTS assays. (**B**) Heatmap of sample activity. (**C**) Distribution of antioxidant activity by material type.

The analysis showed that antioxidant activity in *U. victoris* varied substantially depending on the origin of samples and the type of plant material examined (Table 2 and Table 3; Figure 4). Among natural populations, the highest activity according to the DPPH assay was recorded for sample UV2 (Nilu, 1368.0 ± 51.3 µg/mL), whereas lower activity was observed in UV4 (Sovukbulok, 1803.9 ± 66.2 µg/mL), UV3 (Poyaz, 1992.4 ± 74.1 µg/mL), and especially UV1 (Polgasay, 2397.8 ± 85.6 µg/mL). According to the ABTS assay, the highest activity was also observed in UV4 (Sovukbulok, 471.2 ± 19.4 µg/mL) and UV2 (Nilu, 533.8 ± 21.5 µg/mL), whereas UV3 (Poyaz, 716.2 ± 28.7 µg/mL) and UV1 (Polgasay, 751.3 ± 26.4 µg/mL) showed a lower radical scavenging capacity.

Thus, even within a single species and considering only natural populations, *U. victoris* exhibited pronounced intraspecific heterogeneity, with IC_50_ values varying by approximately 1.75-fold in the DPPH assay and 1.59-fold in the ABTS assay. Such variability is consistent with literature data indicating that, in members of the Amaryllidaceae family, chemical profiles and biological activities depend on genotype, geographic origin, ecological conditions, and plant physiological status [39,40]. More than 636 alkaloids have been described for this family, and these alkaloids, together with associated non-alkaloid metabolites, may exhibit antioxidant, anti-inflammatory, antimicrobial, and neuropharmacological properties [41].

A comparison between natural plants and ex situ cultivated specimens from the botanical garden showed that ex situ cultivation does not necessarily lead to a decline in antioxidant potential. On the contrary, some botanical garden samples demonstrated higher activity than plants from natural coenopopulations. The most illustrative example was sample UV3_BG (Poyaz, 685.7 ± 26.1 µg/mL), which showed the lowest DPPH IC_50_ value among all *U. victoris* samples examined. Samples UV2_BG (Nilu, 1299.3 ± 47.6 µg/mL) and UV4_BG (Sovukbulok, 923.5 ± 34.2 µg/mL) also outperformed most natural populations in the DPPH assay. In the ABTS assay, comparatively high activity was recorded in UV3_BG (Poyaz, 490.0 ± 18.9 µg/mL) and UV1_BG (Polgasay, 538.9 ± 20.7 µg/mL). These findings are in an agreement with current views that introduced and in vitro-derived lines of rare medicinal plants may retain pharmacologically significant properties and can therefore be used as an alternative to harvesting wild-growing raw material [42]. It has already been shown for *U. victoris* and *U. sewerzowii* that in vitro regenerants retain the ability to accumulate valuable alkaloids, including galantamine and lycorine, and such lines are regarded as a tool for conservation of natural populations [29,36,43].

Callus cultures of *U. victoris* are of particular interest. It is important to emphasize that all callus lines were derived exclusively from a single population, UV1 (Polgasay). Despite their common origin, the callus lines differed sharply in antioxidant activity, indicating that the observed variation was determined primarily by medium composition and hormonal treatment rather than by population origin. The highest activity in the ABTS assay was recorded for UV1_V56 (1.84 ± 0.07 µg/mL) and UV1_V5 (4.91 ± 0.20 µg/mL), whereas the lowest DPPH IC_50_ values were also found in UV1_V56 (782.6 ± 28.4 µg/mL) and UV1_V5 (825.6 ± 29.7 µg/mL). Lower activity was observed for UV1_M56 (944.8 ± 33.5 µg/mL) and UV1_V5 + TDZ (955.1 ± 36.4 µg/mL), whereas UV1_V16 (1585.2 ± 58.3 µg/mL) showed markedly weaker activity. This range confirms literature data that in vitro culture can be used to direct the biosynthesis of antioxidant metabolites through optimization of medium composition, plant growth regulators, and stress factors [42,43,44]. Reviews on plant tissue culture indicate that modification of nutrient media and growth regulators represents one of the key strategies for enhancing the production of phenolic and other antioxidant compounds [44].

An additional explanation for these results is that, in plant calli, antioxidant activity is often closely associated with the accumulation of phenolic compounds and flavonoids. In rare and endemic species, changes in the Kin/2.4D ratio have been shown to substantially alter total phenol and flavonoid contents, as well as ABTS and DPPH activity. Increasing the concentration of growth regulators does not always enhance antioxidant potential and may in some cases even reduce it.

In contrast, in vitro-derived bulbs of *U. victoris* were characterized by significantly lower antioxidant activity. For example, UV2_B (Nilu, 8989.0 ± 321.4 µg/mL) and UV3_B (Poyaz, 4409.1 ± 165.2 µg/mL) in the DPPH assay were markedly inferior not only to callus lines but also to most natural and botanical garden samples. This indicates that tissue type strongly influences the antioxidant profile. Similar differences between organs and culture types have been reported in other medicinal species, where in vivo and in vitro organs differed substantially in phenolic compound accumulation and antioxidant activity [40,45].

2. Intraspecific Variability of Antioxidant Activity in *U. sewerzowii*.

**Figure 5:**
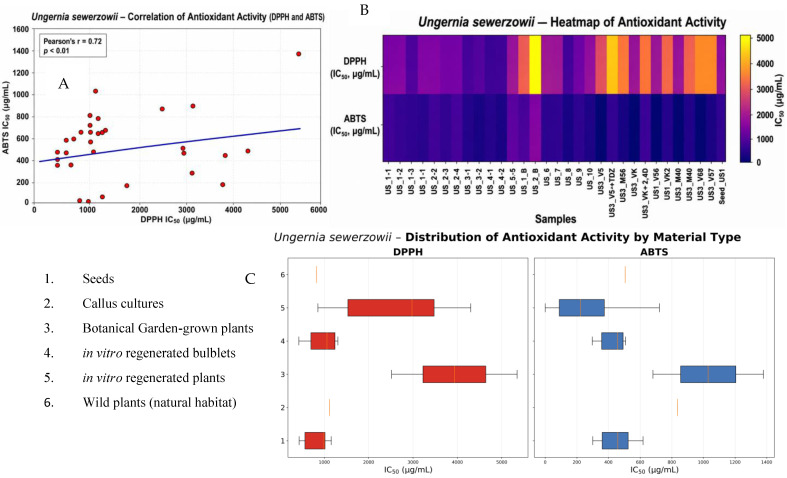
Antioxidant activity of different types of plant material of *Ungernia sewerzowii*. (**A**) Correlation between DPPH and ABTS assays. (**B**) Distribution of antioxidant activity by material type. (**C**) Heatmap of sample activity.

High intraspecific variability in antioxidant activity was also revealed in *U. sewerzowii* (Table 4 and Table 5, Figure 5); however, overall, this species proved to be more active than *U. victoris*. Among natural populations, the best DPPH results were observed in US4 (Beldersay, 433.10 ± 16.1 µg/mL), US4_1 (Beldersay, 442.00 ± 16.5 µg/mL), US3 (Aksarsay, 560.34 ± 20.8 µg/mL), and US3_1 (Aksarsay, 622.00 ± 22.9 µg/mL). Lower activity was recorded in US2 (Gulkamsay, 1016.72 ± 37.5 µg/mL) and US1 (Aksay, 1157.05 ± 42.3 µg/mL). In the ABTS assay, the most pronounced activity was shown by US3_1 (Aksarsay, 300.52 ± 11.4 µg/mL), US4_1 (Beldersay, 335.62 ± 12.8 µg/mL), and US4 (Beldersay, 359.07 ± 13.9 µg/mL), whereas higher IC_50_ values, indicating lower activity, were observed for US1 (Aksay, 594.25 ± 23.1 µg/mL) and US2_3 (Gulkamsay, 618.47 ± 24.5 µg/mL).

The intraspecific range in *U. sewerzowii* was approximately 2.67-fold in the DPPH assay and 2.06-fold in the ABTS assay, which is even greater than that observed in *U. victoris*. This greater intraspecific variation is fully consistent with the literature on Amaryllidaceae, where pronounced chemodiversity is emphasized both among species and within individual taxa [40,41]. Reviews of this family indicate that alkaloid composition and associated phenolic acids may vary considerably under the influence of taxonomic, geographic, and ecological factors [40].

Botanical garden plants of *U. sewerzowii* also exhibited high activity, and some samples even outperformed natural populations. The most representative was US3_BG (Aksarsay, 428.80 ± 15.7 µg/mL in DPPH and 297.02 ± 11.2 µg/mL in ABTS), making it one of the most promising samples in the entire dataset. Sample US2_BG_1 (Gulkamsay, 1058.81 ± 38.7 µg/mL; 357.21 ± 13.9 µg/mL) also demonstrated pronounced antioxidant activity. This confirms that botanical garden collections may serve not only as a means of conserving endangered species but also as a source of standardized biologically active raw material. This conclusion is consistent with the general concept that in vitro and ex situ systems are regarded as environmentally sustainable sources of valuable metabolites in threatened species [38,42].

For *U. sewerzowii* callus cultures, it should again be emphasized that all lines were derived exclusively from a single population, US3 (Aksarsay). In this case, the effects of nutrient medium and plant growth regulators were particularly pronounced. The highest antioxidant activity in the ABTS assay was recorded for US3_VK (0.42 ± 0.02 µg/mL), followed by US3_V56 (5.35 ± 0.21 µg/mL). In the DPPH assay, the best callus values were also observed for US3_VK (857.65 ± 31.7 µg/mL) and US3_V56 (1002.50 ± 36.9 µg/mL). At the same time, the addition of certain growth regulator combinations led to a sharp decline in activity: US3_VK + 2.4D (3162.03 ± 116.7 µg/mL; 721.75 ± 28.4 µg/mL) and US3_V5 + TDZ (4301.23 ± 158.4 µg/mL; 387.19 ± 14.6 µg/mL) were substantially less active.

This contrast is especially important for interpreting the results, as it demonstrates that, in *U. sewerzowii*, the very fact of callusogenesis does not guarantee a high antioxidant potential; rather, the decisive factor is the specific hormone–medium combination. This is fully consistent with literature data indicating that the efficiency of secondary metabolite biosynthesis in in vitro systems is determined by a combination of biological, chemical, and physical factors, including plant growth regulators, stress signals, and elicitors [42,44]. In *Leucojum aestivum* cultures, even factors such as light, temperature, and other stressors have been shown to substantially alter alkaloid biosynthesis and antioxidant responses [45,46].

3. Comparative Analysis of *U. victoris* and *U. sewerzowii*.

A comparison of the two species showed that, on average, natural populations of *U. sewerzowii* exhibited higher antioxidant activity than those of *U. victoris*. In the DPPH assay, the average IC_50_ values for natural samples of *U. victoris* were approximately 1890 µg/mL, whereas for *U. sewerzowii* they were about 815 µg/mL. In the ABTS assay, the differences were also clear: approximately 618 µg/mL for *U. victoris* versus approximately 454 µg/mL for *U. sewerzowii*. Therefore, *U. sewerzowii* can be regarded as the species with the higher intrinsic antioxidant potential.

From the standpoint of the literature, such interspecific differences are expected. The Amaryllidaceae family is characterized by pronounced chemical heterogeneity, and even closely related species may differ substantially in the composition of alkaloids, phenolic acids, and associated biological effects [41,45,47,48,49]. Reviews of the family emphasize that the pharmacological value of Amaryllidaceae species is determined not only by the presence of galantamine, lycorine, or other well-known alkaloids, but also by a wide spectrum of accompanying compounds with antioxidant activity.

At the same time, the transition from natural plants to biotechnological systems complicates the observed patterns. In *U. victoris*, callus lines were generally characterized by a more stable increase in antioxidant activity, especially in the ABTS assay. In contrast, *U. sewerzowii* showed substantially greater variability between the most and least active callus lines. This suggests that *U. victoris* responds more predictably to callusogenesis by increasing antioxidant potential, whereas in *U. sewerzowii* the achievement of maximum effect depends more strongly on precise optimization of cultivation conditions.

4. Influence of Culture Media and Plant Growth Regulators on Antioxidant Activity.

The results of the present study clearly demonstrate that medium composition and phytohormonal regime are among the key factors determining the antioxidant activity of callus cultures. In *U. victoris*, the most effective variants were UV1_V56 (782.6 ± 28.4 µg/mL; 1.84 ± 0.07 µg/mL) and UV1_V5 (825.6 ± 29.7 µg/mL; 4.91 ± 0.20 µg/mL), whereas UV1_V16 (1585.2 ± 58.3 µg/mL; 25.35 ± 1.1 µg/mL) and UV1_VK + 2,4-D (1142.2 ± 41.7 µg/mL; 31.38 ± 1.4 µg/mL) were less effective. In *U. sewerzowii*, the strongest effect was observed for US3_VK (857.65 ± 31.7 µg/mL; 0.42 ± 0.02 µg/mL) and US3_V56 (1002.50 ± 36.9 µg/mL; 5.35 ± 0.21 µg/mL), whereas the addition of TDZ or strengthening of the auxin component was accompanied by deterioration in antioxidant parameters.

The literature generally supports such a dependence. Reviews on plant tissue culture show that the production of phenolic compounds and antioxidant activity depend on the explant type, medium composition, the nature of auxins and cytokinins, and the intensity of the cellular stress response. It has been established that Kin, 2.4D, BAP, TDZ, and other growth regulators may either enhance or suppress the synthesis of secondary metabolites, while the optimal combinations for morphogenesis do not always coincide with the optimum for accumulation of antioxidant compounds.

From a practical standpoint, this means that the development of a biotechnological platform for *Ungernia* species requires dual optimization: one aimed at maximizing biomass production and another aimed at maximizing the accumulation of antioxidant-active compounds. This is particularly important for Amaryllidaceae species, since in vitro conditions, light, and stress signals are known to simultaneously affect both alkaloid content and the pool of phenolic compounds [42,45,46].

5. Correlation of Antioxidant Activity Determined by the DPPH and ABTS Assays.

The positive correlation revealed between antioxidant activity values determined by the DPPH and ABTS assays indicates the consistency of the analytical approaches used and confirms the reliability of the obtained results (Figure 4 and Figure 5). This relationship suggests that the compounds responsible for antioxidant activity are capable of effectively interacting with both the DPPH radical and the ABTS radical cation.

Of particular importance is the fact that members of the family *Amaryllidaceae*, including *U. victoris* and *U. sewerzowii*, are sources of biologically active alkaloids, among which galantamine plays a key role. Galantamine, a benzazepine-type alkaloid widely used in the treatment of Alzheimer’s disease, exhibits not only acetylcholinesterase inhibitory activity but also moderate antioxidant properties. In this context, it may be assumed that part of the observed antioxidant activity of the studied samples is associated with the accumulation of Amaryllidaceae alkaloids, including galantamine.

At the same time, the high degree of correlation between the DPPH and ABTS assays suggests that antioxidant activity is not attributable to a single compound, but rather to a complex of secondary metabolites. In addition to alkaloids, phenolic compounds, flavonoids, and other antioxidants may make a substantial contribution. This is particularly evident in callus cultures, which are characterized by extremely low IC_50_ values in the ABTS assay, indicating the accumulation of hydrophilic antioxidant compounds under in vitro conditions.

The observed variability in antioxidant activity among different types of plant material likely reflects differences in the biosynthesis of secondary metabolites. It is well established that in vitro conditions and the application of growth regulators such as 2.4D, TDZ, and Kin can substantially alter metabolic fluxes and stimulate the biosynthesis of Amaryllidaceae alkaloids, including galantamine.

In contrast, in vitro-derived bulbs were characterized by relatively low antioxidant activity, which may indicate insufficient accumulation of secondary metabolites at this developmental stage. Samples from natural populations and botanical garden plants occupied an intermediate position, reflecting a more stable metabolic status under natural and introduced conditions.

**Figure 6:**
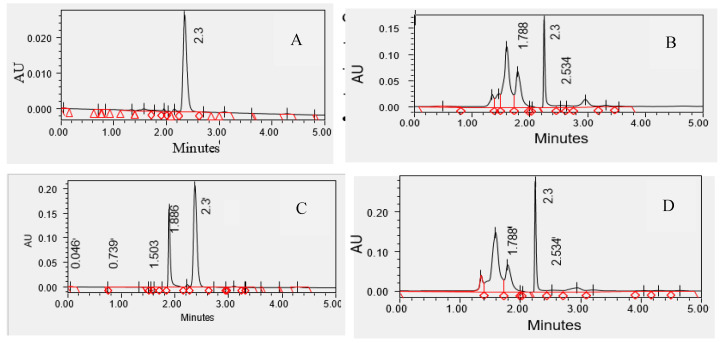
HPLC chromatograms of galantamine standard and callus extracts of *Ungernia victoris*. (**A**) Galantamine standard (10 mg/L). (**B**) Callus culture grown on V5 medium (2,4-D 0.5 mg/L + BAP 0.5 mg/L). (**C**) Callus culture grown on V56 medium (2,4-D 0.5 mg/L + Kin 0.5 mg/L). (**D**) Callus culture grown on V57 medium (2,4-D 0.5 mg/L + Kin 1.0 mg/L). Galantamine was identified by comparison of retention times with the authentic standard (RT ≈ 2.3 min).

To support the interpretation of antioxidant activity, a preliminary phytochemical characterization of the extracts was performed using HPLC analysis. The chromatographic profiles revealed the presence of multiple peaks, indicating the complex composition of the studied samples. Representative HPLC chromatograms illustrate the chemical profile of the extracts and confirm the presence of galantamine as a characteristic alkaloid of *Ungernia victoris* samples (Figure 6).

The presence of galantamine is consistent with literature data and confirms the accumulation of Amaryllidaceae alkaloids in both natural and in vitro-derived materials. In addition to galantamine, both species are known to contain lycorine, pancratinine, narwedine, tazettine, ungerine and other Amaryllidaceae alkaloids, which may also contribute directly or indirectly to the antioxidant properties of the extracts.

Detailed HPLC data on galantamine content in different samples are provided in the Supplementary Materials (Table S2). Comprehensive qualitative and quantitative profiling of individual alkaloids using advanced analytical techniques such as LC–MS or NMR is beyond the scope of the present study and will be reported in a separate investigation.

Thus, the antioxidant activity of *U. sewerzowii* is determined by the combined action of Amaryllidaceae alkaloids, including galantamine, as well as other classes of secondary metabolites. The high correlation between the DPPH and ABTS assays confirms the presence of multifunctional antioxidant compounds, whereas the pronounced activity of callus cultures highlights their potential as a biotechnological platform for the production of biologically active substances, including galantamine.

6. Significance of the Results for Pharmaceutical Production and Species Conservation.

The pharmaceutical relevance of the present results is defined by the fact that both species of the genus *Ungernia* represent valuable sources of biologically active compounds of the family *Amaryllidaceae*. Galantamine is a clinically used acetylcholinesterase inhibitor for the treatment of Alzheimer’s disease, whereas its industrial production from plant raw material has historically been associated with low yield and depletion of natural resources. The literature clearly indicates that galantamine is obtained from several *Amaryllidaceae* species, including *U. victoris*; however, both field harvesting and industrial extraction remain costly and not always sustainable [16,26,36,39,49,50].

Therefore, the results of the present study have a dual applied significance. First, they identify the populations and lines that are most promising as sources of antioxidant-active raw material. For *U. victoris*, these include primarily UV2 (Nilu, 1368.0 ± 51.3 µg/mL), UV4 (Sovukbulok, 471.2 ± 19.4 µg/mL in the ABTS assay), UV3_BG (Poyaz, 685.7 ± 26.1 µg/mL), and the callus lines UV1_V56 (782.6 ± 28.4 µg/mL; 1.84 ± 0.07 µg/mL) and UV1_V5 (825.6 ± 29.7 µg/mL; 4.91 ± 0.20 µg/mL). For *U. sewerzowii*, the most promising materials are US4 (Beldersay, 433.10 ± 16.1 µg/mL), US3_1 (Aksarsay, 300.52 ± 11.4 µg/mL in the ABTS assay), US3_BG (Aksarsay, 428.80 ± 15.7 µg/mL; 297.02 ± 11.2 µg/mL), and the callus lines US3_VK (857.65 ± 31.7 µg/mL; 0.42 ± 0.02 µg/mL) and US3_V56 (1002.50 ± 36.9 µg/mL; 5.35 ± 0.21 µg/mL).

Second, the results confirm that conservation and sustainable use of these species should be associated not only with protection of natural populations, but also with the development of ex situ and in vitro systems. For rare medicinal plants, this is considered one of the most justified strategies, as it reduces pressure on natural communities while preserving access to valuable metabolic resources. For *Amaryllidaceae*, it has already been shown that tissue cultures and in vitro regenerants can serve both as an alternative route for the production of biologically active compounds and as a tool for the conservation of endangered species [38,42].

## 4. Conclusions

The obtained data demonstrate that antioxidant activity in *U. victoris* and *U. sewerzowii* is determined by the combined influence of three levels of factors: (1) population origin, (2) type of biological material, and (3) culture medium composition and phytohormonal regime under in vitro conditions.

The literature confirms that such multilevel variability is typical of Amaryllidaceae species and of medicinal plant tissue cultures in general [38,39,50]. Intraspecific chemodiversity, dependence on cultivation conditions, and the possibility of directed enhancement of secondary metabolite biosynthesis make both studied species promising objects for pharmaceutical biotechnology as well as conservation programs. Although the antioxidant activity of the investigated samples was lower than that of the reference antioxidant, ascorbic acid, substantial differences were observed among natural populations, in vitro-derived materials, and callus cultures.

At the same time, the results help to define practical priorities: for *U. victoris*, the most promising antioxidant platform is represented by callus lines derived from UV1 on media V56 and V5; for *U. sewerzowii*, the highest natural antioxidant activity was observed in populations US3 and US4, whereas among callus cultures, the most promising were the lines derived from US3 on media VK and V56. These findings indicate that selected callus cultures may serve as a sustainable source of antioxidant compounds and provide a basis for further optimization of biotechnological production systems.

## 5. Patents

1. Mustafina, F.U.; Juraeva, H.K.; Jamalova, D.N.; Kurbaniazova, G.T.; Abdinazarov, S.Kh. Method for Microclonal Propagation of *Ungernia sewerzowii* (Regel) B.Fedtsch. (Amaryllidaceae J.St.-Hil.) (Variants). Patent No. IAP 8178, registered in the State Register of Inventions of the Republic of Uzbekistan on 8 August 2025.

2. Juraeva, H.K.; Mustafina, F.U.; Jamalova, D.N.; Kurbaniazova, G.T.; Abdinazarov, S.Kh. Method for Microclonal Propagation of *Ungernia victoris* Vved. ex Artjush. (Amaryllidaceae J.St.-Hil.). Patent No. IAP 8179, registered in the State Register of Inventions of the Republic of Uzbekistan on 8 August 2025.

## Figures and Tables

**Table 1:** Sampling sites of *Ungernia victoris* and *Ungernia sewerzowii* within their natural habitats in the Republic of Uzbekistan.

Species	Population	Sampling Site	Collection Date	Coordinates	Altitude (m a.s.l.)
*U. victoris*	UV1	Polgasay River valley, vicinity of the Sangardak waterfall, Hissar Range, Surkhandarya region, Uzbekistan	10 April 2023	38°23′44.9″ N, 67°35′12.8″ E	1838
	UV2	Vicinity of Nilu village, Sariosiyo district, Hissar Range, Pamir-Alai, Surkhandarya region, Uzbekistan	15 April 2023	38°22′21″ N, 67°34′13″ E	1350
	UV3	Vicinity of Poyaz village, Sariosiyo district, Hissar Range, Pamir-Alai, Surkhandarya region, Uzbekistan	17 April 2023	38°23′01″ N, 67°33′42″ E	1450
	UV4, UV5	Sovukbulak River valley, approximately 10 km from Padang village, Hissar Range, Pamir-Alai, Surkhandarya region, Uzbekistan	17 April 2023	38°24′17.6″ N, 67°31′16.7″ E	1400
*U. sewerzowii*	US1	Aksay and Katta-Koksay river valleys, Greater Chimgan area, Tashkent region, Western Tien Shan, Uzbekistan	8 April 2020	41°30′43.8″ N, 70°03′03.1″ E	1900
	US2	Gulkamsay River valley, Chatkal Range, Western Tien Shan, Tashkent region, Uzbekistan	12 April 2020	41°48′14″ N, 70°05′26″ E	1700
	US3	Aksarsay River valley, vicinity of Nanay village, Pskem Range, Western Tien Shan, Tashkent region, Uzbekistan	25 June 2021	41°41′26.1″ N, 70°14′04.1″ E	1500
	US4, US5	Beldersay River valley, vicinity of the meteorological station, Chatkal Range, Western Tien Shan, Tashkent region, Uzbekistan	7 June 2021	41°28′35.3″ N, 69°58′32.9″ E	2275

**Table 2:** Antioxidant activity of different types of plant material of *Ungernia victoris* obtained from natural populations of the Hissar Range (Pamir-Alai mountain system, Surkhandarya region, Uzbekistan) and from in vitro cultures.

Material Type	Sample Code	Origin	Extract Yield (%)	DPPH IC_50_ (µg/mL)	ABTS IC_50_ (µg/mL)
Plants from nature	UV1	Polgasay	10.5 ± 0.4	2397.8 ± 85.6	751.3 ± 26.4
	UV2	Nilu	7.7 ± 0.3	1368.0 ± 51.3	533.8 ± 21.5
	UV3	Poyaz	7.8 ± 0.3	1992.4 ± 74.1	716.2 ± 28.7
	UV4	Sovukbulok	3.1 ± 0.1	1803.9 ± 66.2	471.2 ± 19.4
in vitro regenerated plants	UV5	Polgasay	8.6 ± 0.3	1542.8 ± 57.8	823.8 ± 33.5
in vitro regenerated bulbs	UV1_B	Polgasay	1.3 ± 0.05	–	–
	UV2_B	Nilu	7.2 ± 0.3	8989.0 ± 321.4	1580.2 ± 64.2
	UV3_B	Poyaz	5.3 ± 0.2	4409.1 ± 165.2	1496.7 ± 55.1
	UV4_B	Sovukbulok	7.1 ± 0.3	3556.1 ± 134.6	1081.0 ± 41.3
Plants grown in Botanical Garden	UV1_BG	Polgasay	4.3 ± 0.2	1580.5 ± 59.8	538.9 ± 20.7
	UV1_BG_1	Polgasay	6.3 ± 0.3	2389.9 ± 90.1	591.3 ± 24.2
	UV2_BG	Nilu	11.0 ± 0.4	1299.3 ± 47.6	537.3 ± 19.5
	**UV3_BG**	Poyaz	9.8 ± 0.4	**685.7 ± 26.1**	**490.0 ± 18.9**
	UV4_BG	Sovukbulok	9.0 ± 0.3	923.5 ± 34.2	603.8 ± 23.8
	UV4_BG_1	Sovukbulok	15.5 ± 0.6	2297.7 ± 83.6	729.7 ± 27.9
Callus tissue	UV1_V5 + TDZ 0.5 mg/L	Vch ^1^	8.4 ± 0.3	955.1 ± 36.4	2.15 ± 0.08
	UV1_VK + 2.4D 1.0	Vch ^1^	10.1 ± 0.4	1142.2 ± 41.7	31.38 ± 1.4
	UV1_M56	MS ^2^	6.6 ± 0.2	944.8 ± 33.5	53.53 ± 2.1
	UV1_V57	Vch ^1^	13.1 ± 0.5	1465.5 ± 52.8	11.33 ± 0.5
	UV1_V5	Vch ^1^	15.2 ± 0.6	825.6 ± 29.7	4.91 ± 0.2
	**UV1_V56**	Vch ^1^	18.8 ± 0.7	**782.6 ± 28.4**	**1.84 ± 0.07**
	UV1_V16	Vch ^1^	11.4 ± 0.4	1585.2 ± 58.3	25.35 ± 1.1
Seeds	UV1_S	Polgasay	14.8 ± 0.5	–	4413.3 ± 170.5
	UV4_S	Sovukbulok	16.2 ± 0.6	3595.1 ± 135.2	3538.9 ± 141.7

Lower IC_50_ values correspond to higher antioxidant activity. ^1^ Vollosovich et al. medium (Vch); ^2^ Murasige and Skoog medium (MS). Samples with the highest antioxidant activity are highlighted in bold. Positive control: IC_50_ of ascorbic acid—DPPH 17.21 µg/mL; ABTS 8.33 µg/mL.

**Table 3:** Mean values of antioxidant activity of callus cultures of *Ungernia victoris*.

Medium	DPPH IC_50_ (µg/mL)	ABTS IC_50_ (µg/mL)
Murasige and Skoog medium	1264.97 ± 452.86	39.44 ± 19.93
Vollosovich et al. medium	1034.18 ± 278.74	10.32 ± 12.37

**Table 4:** Antioxidant activity of different types of plant material of *Ungernia sewerzowii* obtained from natural populations of the Western Tien Shan (Tashkent region, Uzbekistan) and from in vitro cultures.

Material Type	Sample Code	Origin	Extract Yield (%)	DPPH IC_50_ (µg/mL)	ABTS IC_50_ (µg/mL)
Nature populations	US1	Aksay	14.82 ± 0.55	1157.05 ± 42.3	594.25 ± 23.1
	US1_1	Aksay	8.77 ± 0.31	1153.21 ± 41.8	486.07 ± 18.9
	US1_2	Aksay	3.29 ± 0.12	566.88 ± 21.4	458.05 ± 17.6
	US2	Gulkamsay	13.21 ± 0.49	1016.72 ± 37.5	434.09 ± 16.5
	US2_1	Gulkamsay	14.52 ± 0.54	1000.10 ± 36.2	549.79 ± 21.3
	US2_2	Gulkamsay	13.98 ± 0.52	1015.12 ± 37.0	499.46 ± 19.7
	US2_3	Gulkamsay	13.03 ± 0.46	994.37 ± 35.9	618.47 ± 24.5
	US3	Aksarsay	2.94 ± 0.11	560.34 ± 20.8	361.79 ± 14.2
	US3_1	Aksarsay	6.34 ± 0.23	622.00 ± 22.9	300.52 ± 11.4
	US4	Beldersay	9.39 ± 0.35	**433.10 ± 16.1**	359.07 ± 13.9
	US4_1	Beldersay	7.74 ± 0.29	**442.00 ± 16.5**	335.62 ± 12.8
in vitro regenerated plants	US_5	Beldersay	10.16 ± 0.37	1115.25 ± 40.6	835.66 ± 31.2
in vitro regenerated bulbs	US1_B	Aksay	4.81 ± 0.18	2516.50 ± 91.3	681.43 ± 26.5
	US2_B	Gulkamsay	3.33 ± 0.12	5346.56 ± 196.4	1379.67 ± 53.6
	US3_B	Aksarsay	7.37 ± 0.27	–	–
	US4_B	Beldersay	4.44 ± 0.16	–	–
Plants grown in Botanical Garden	US1_BG	Aksay	6.30 ± 0.23	1241.16 ± 45.2	492.65 ± 19.1
	US2_BG	Gulkamsay	6.81 ± 0.25	1305.33 ± 48.6	508.61 ± 20.4
	US2_BG_1	Gulkamsay	9.23 ± 0.34	1058.81 ± 38.7	357.21 ± 13.9
	**US3_BG**	Aksarsay	3.43 ± 0.12	**428.80 ± 15.7**	**297.02 ± 11.2**
	US4_BG	Beldersay	5.67 ± 0.21	698.55 ± 25.9	457.37 ± 17.5
Callus cultures	US3_V5	Vch ^1^	15.49 ± 0.57	1796.60 ± 66.2	139.60 ± 5.2
	US3_V5 + TDZ	Vch ^1^	14.66 ± 0.54	4301.23 ± 158.4	387.19 ± 14.6
	US3_M56	MS ^2^	12.45 ± 0.46	3131.77 ± 115.2	222.91 ± 8.4
	US3_VK1	Vch ^1^	15.89 ± 0.59	1267.62 ± 46.1	39.18 ± 1.5
	US3_VK1 + 2.4D 1.0	Vch ^1^	21.90 ± 0.81	3162.03 ± 116.7	721.75 ± 28.4
	**US3_V56**	Vch ^1^	13.41 ± 0.49	**1002.50 ± 36.9**	**5.35 ± 0.21**
	US1_V56	Vch ^1^	17.08 ± 0.63	2977.06 ± 110.4	359.93 ± 13.8
	**US3_VK1**	Vch ^1^	15.91 ± 0.59	**857.65 ± 31.7**	**0.42 ± 0.02**
	US3_M40	MS ^2^	28.79 ± 1.06	2953.43 ± 108.5	395.60 ± 15.4
	US3_V68	Vch ^1^	11.36 ± 0.42	3785.91 ± 139.8	164.32 ± 6.1
	US3_V57	Vch ^1^	13.04 ± 0.48	3833.24 ± 141.5	343.39 ± 13.2
Seeds	US1_S	Aksay	6.36 ± 0.23	824.22 ± 30.1	506.30 ± 19.4

Lower IC_50_ values correspond to higher antioxidant activity. ^1^ Vollosovich et al. medium (Vch); ^2^ Murasige and Skoog medium (MS). Samples with the highest antioxidant activity are highlighted in bold. Positive control: IC_50_ of ascorbic acid—DPPH 17.21 µg/mL; ABTS 8.33 µg/mL.

**Table 5:** Mean values of antioxidant activity of callus cultures of *Ungernia sewerzowii*.

Medium	DPPH IC_50_ (μg/mL)	ABTS IC_50_ (μg/mL)
Murasige and Skoog medium	3042.60 ± 126.11	309.26 ± 122.11
Vollosovich et al. medium	2553.76 ± 1335.51	240.13 ± 236.49

## Data Availability

The data presented in this study are available within the article and Supplementary Materials. Additional data are available from the corresponding author upon reasonable request.

## Supplementary Materials

The following supporting information can be downloaded at: https://www.mdpi.com/article/10.3390/antiox15060763/s1, Table S1: Raw data of antioxidant activity (DPPH and ABTS assays) and extract yield; Table S2: HPLC analysis of galantamine content in *Ungernia victoris* samples.

## Abbreviations

The following abbreviations are used in this manuscript:

ABTS	2,2′-azino-bis(3-ethylbenzothiazoline-6-sulfonic acid)
BAP	6-benzylaminopurine
BG	Botanical Garden
DPPH	2,2-diphenyl-1-picrylhydrazyl
IAA	Indole-3-acetic acid
IC_50_	Half maximal inhibitory concentration
Kin	Kinetin
MS	Murasige and Skoog medium
NAA	α-naphthaleneacetic acid
ROS	Reactive oxygen species
SD	Standard deviation
TDZ	Thidiazuron
UV	*Ungernia victoris*
US	*Ungernia sewerzowii*
Vch	Vollosovich et al. medium
